# Applying Robotic Process Automation to Monitor Business Processes in Hospital Information Systems: Mixed Method Approach

**DOI:** 10.2196/59801

**Published:** 2025-03-07

**Authors:** Adam Park, Se Young Jung, Ilha Yune, Ho-Young Lee

**Affiliations:** 1 Office of eHealth Research and Business Seoul National University Bundang Hospital Seongnam-si Republic of Korea; 2 Department of Family Medicine College of Medicine Seoul National University Seoul Republic of Korea; 3 Department of Nuclear Medicine College of Medicine Seoul National University Seoul Republic of Korea

**Keywords:** robotic process automation, RPA, electronic medical records, EMR, system monitoring, health care information systems, user-centric monitoring, performance evaluation, business process management, BPM, healthcare technology, mixed methods research, process automation in health care

## Abstract

**Background:**

Electronic medical records (EMRs) have undergone significant changes due to advancements in technology, including artificial intelligence, the Internet of Things, and cloud services. The increasing complexity within health care systems necessitates enhanced process reengineering and system monitoring approaches. Robotic process automation (RPA) provides a user-centric approach to monitoring system complexity by mimicking end user interactions, thus presenting potential improvements in system performance and monitoring.

**Objective:**

This study aimed to explore the application of RPA in monitoring the complexities of EMR systems within a hospital environment, focusing on RPA’s ability to perform end-to-end performance monitoring that closely reflects real-time user experiences.

**Methods:**

The research was conducted at Seoul National University Bundang Hospital using a mixed methods approach. It included the iterative development and integration of RPA bots programmed to simulate and monitor typical user interactions with the hospital’s EMR system. Quantitative data from RPA process outputs and qualitative insights from interviews with system engineers and managers were used to evaluate the effectiveness of RPA in system monitoring.

**Results:**

RPA bots effectively identified and reported system inefficiencies and failures, providing a bridge between end user experiences and engineering assessments. The bots were particularly useful in detecting delays and errors immediately following system updates or interactions with external services. Over 3 years, RPA monitoring highlighted discrepancies between user-reported experiences and traditional engineering metrics, with the bots frequently identifying critical system issues that were not evident from standard component-level monitoring.

**Conclusions:**

RPA enhances system monitoring by providing insights that reflect true end user experiences, which are often overlooked by traditional monitoring methods. The study confirms the potential of RPA to act as a comprehensive monitoring tool within complex health care systems, suggesting that RPA can significantly contribute to the maintenance and improvement of EMR systems by providing a more accurate and timely reflection of system performance and user satisfaction.

## Introduction

The cutting-edge technologies in health care demand intense transformations in electronic medical records (EMRs) by adopting new services, including artificial intelligence (AI)–driven predictions, Internet of Things (IoT) supports, telemedicine interfaces, and cloud-based open services [1-5]. These transformations imply a rapid growth in complexity and necessitate process re-engineering in EMR systems. Complexity management in dynamic health care environments has been studied by researchers who perceived the uncertainty and unpredictability inherent to electronic health record (EHR) systems [6-8].

One significant limitation is that the actual end user experience is gradually neglected as the performance metric “improves.” The focus is predominantly on component-level analyses, allowing engineers to divide labor and concentrate efforts. This approach can create gaps between the perceptions of end users and engineers in measuring system performance.

The crux of the problem lies in revealing the “interrelatedness of components” in EHRs, meaning that the mutual influences among services, modules, and business processes should be measured and tracked [9]. No single standardized model has yet emerged, leaving various complex approaches for identifying interacting components [10]. One contribution to identifying the degree of interrelatedness is end-to-end performance monitoring, which suggests measuring system complexity from an external, comprehensive, and user-centric perspective. Application performance monitoring (APM), for example, measures transaction latency from user applications, hence complementing the efforts of engineers focused on small, manageable, and self-contained components [11]. However, such approaches, sensitive to data interoperability and dynamically changing environments [12], have been too costly to be widely adopted in health care where the focus is on business re-engineering rather than system maintenance and monitoring [13].

Robotic process automation (RPA) shows potential competitiveness in external monitoring with functions focusing on human-mimicking features. A robot, in this sense, is an automated assistant residing in a computing environment, performing tasks that imitate those of people controlling other applications, usually upon an operating system [14]. The robotic metaphor thus includes 3 internal features corresponding to the human abilities of perceiving, deciding, and inputting, which have all been revolutionized by AI-based cognitive computing [15] and the three internal features are (1) an RPA bot interprets symbols on the graphical user interface (GUI); (2) an RPA bot determines actions to comply with predefined business rules; and (3) an RPA bot inputs signals into applications, mimicking those from peripheral devices used by end users.

These abilities allow the automated agents to perform manual and labor-intensive tasks, automating administrative tasks in back offices [16], including dashboard monitoring, low-judgment auditing tasks, and accounting process automation in finance, banking, and IT services [14,17-19]. Therefore, the implication of RPA in complexity management is end-to-end performance monitoring that reflects the experiences of end users by simulating their activities and freeing engineers from excessive feedback [20]. A “robot” can fulfill external monitoring with an outside-in approach that witnesses the same glitches end users perceive [21].

RPA, with its “build above system” features, has the potential to combine with business process management (BPM) and overcome the intrinsic limitation that systems should be re-engineered. BPM has succeeded in establishing patterns for business process integrations in health care but has not been effective at guiding process monitoring for risk management [22,23]. Continuous reforms in health care organizations have been mandated for their operational success and patient-focused process improvements [24,25]. Thus, the expected synergy between the 2 methodologies is that RPA enhances costly process automation, while BPM provides the holistic perspective of detecting, auditing, and optimizing business processes [26].

This study explored the potential of RPA in complex EMR systems with its role as “a canary in a coal mine” [11], presenting generalizable findings from a 3-year project at Seoul National University Bundang Hospital (SNUBH).

The paper posits RPA as a means to bridge the gap between the limitations of both component-level system monitoring and application-level monitoring. The former, with its engineering-centric approach, often fails to align with the end users’ experiences, as system monitoring reports may not accurately reflect what end users actually encounter. The latter, while focused on the application level, cannot offer a universal solution due to its lack of scalability and the high costs associated with performance analyses. RPA, in contrast, facilitates a user-centric perspective by designing bots that act as representative users within the hospital, performing the most essential and shared activities. Consequently, any failures or performance degradations observed by the RPA bots are indicative of significant issues within the system, potentially leading to a systemic collapse in the worst case.

The analysis revealed significantly enhanced capabilities of RPA bots beyond initial expectations. Much like a meticulous human tester, RPA bots frequently identified errors immediately following software deployments and detected failures in external services outside the expertise of in-house developers. However, certain challenges hindered RPA from becoming a universal solution for monitoring services. This conclusion is supported by both quantitative data from RPA process outputs and qualitative insights obtained from interviews with hospital practitioners.

## Methods

### Ethical Considerations

#### Human Subject Ethics Review Approvals or Exemptions

This study was conducted in accordance with the ethical guidelines of the institutional review board (IRB) of SNUBH (approval number SNUBH-IRB-2021-004). The research involved secondary analysis of anonymized patient data extracted from hospital information systems. In addition, our study included user interviews, for which individual approvals were obtained in accordance with the IRB guidelines. Due to the anonymization of patient data, the IRB granted an exemption from requiring individual informed consent for the secondary data analysis, as per institutional policies and national regulations.

#### Informed Consent

As the study used pre-existing anonymized data, informed consent was waived by the IRB. The original data collection process ensured that all participants consented to the use of their data for research purposes, which was documented in the hospital’s consent forms. For the user interviews conducted as part of the study, individual informed consent was obtained from all participants, aligning with the guidance provided by the IRB. The scope of the consent for both the secondary data and interviews included future secondary analyses, adhering to institutional policies.

#### Privacy and Confidentiality

All patient data were anonymized before analysis, with direct identifiers removed and indirect identifiers encrypted. Access to the data was restricted to authorized personnel only, and secure servers were used to store and process the information. Data-sharing protocols adhered to the guidelines stipulated by the Health Insurance Portability and Accountability Act (HIPAA).

#### Compensation Details

No compensation was provided to participants, as this study exclusively involved the analysis of pre-existing, anonymized data and user interviews. The nature of the research did not require any direct monetary engagement with participants.

### Project Background

SNUBH management expressed strong interest in integrating monitoring services to identify continuous performance degradations from the end user’s perspective. The Healthcare Information and Management Systems Society in the United States has mandated health care organizations, especially those that have achieved Stage 7 certification, to detect and prepare for unexpected system downtime [21,27]. Furthermore, internal reports from users regarding performance degradations were often overlooked by engineers due to the absence of appropriate analysis tools. Application-level monitoring frameworks, such as APM [28], were not favored by the management mainly, primarily due to high reengineering costs associated with re-engineering [12].

In the meantime, hospital managers found themselves redundantly checking the performance of business processes in unsystematic ways. The requirement to manually perform these tasks led to intermittent execution and a lack of precision. Consequently, hospital management anticipated that RPA’s mechanism could supplant the labor wasted on manual testing to monitor performance regression [29].

### Robotic Process Automation Integration

#### Iterative Development Process

A task force team engaged in an iterative process to implement system-monitoring RPA bots. The objectives were to reliably simulate business workflows on the hospital’s production systems and to establish an organizational protocol for responding to detected performance anomalies.

The proof of Concept process, spanning from January to July 2020, involved repeating cycles from developing requirements to implementing prototypes. This approach was adopted because of the belief that establishing business requirements for a novel methodology should be carried out with meticulous attention to detail. To broaden their understanding of the field, the administration contracted with different RPA third-party vendors at each iteration. Alongside the software prototype development, managers also put in place organizational protocols to react to alerts, with responses tailored to the severity level of the incident.

The development of RPA bots followed an iterative process to address challenges specific to monitoring EMR systems. Frequent user interface (UI) updates initially made it difficult for bots to recognize dynamic elements, but this was resolved by implementing advanced pattern recognition algorithms and adaptive learning techniques to enable automatic adjustments to UI changes. Early versions of the bots also relied on timer-based execution, which caused inefficiencies when dealing with variable system latencies. To address this, event-driven configurations were introduced, allowing the bots to respond dynamically to system triggers.

Ensuring stable performance across diverse environments was another challenge. Extensive pilot testing and iterative adjustments refined the bots’ logic, enabling them to operate reliably in varied setups. Incorporating feedback from multiple departments presented additional complexity, but this was streamlined by centralizing feedback and conducting regular cross-functional meetings to align bot functionality with operational needs.

This iterative approach significantly improved bot performance and adaptability for broader health care applications.

#### Technical Environment

**Figure 1:**
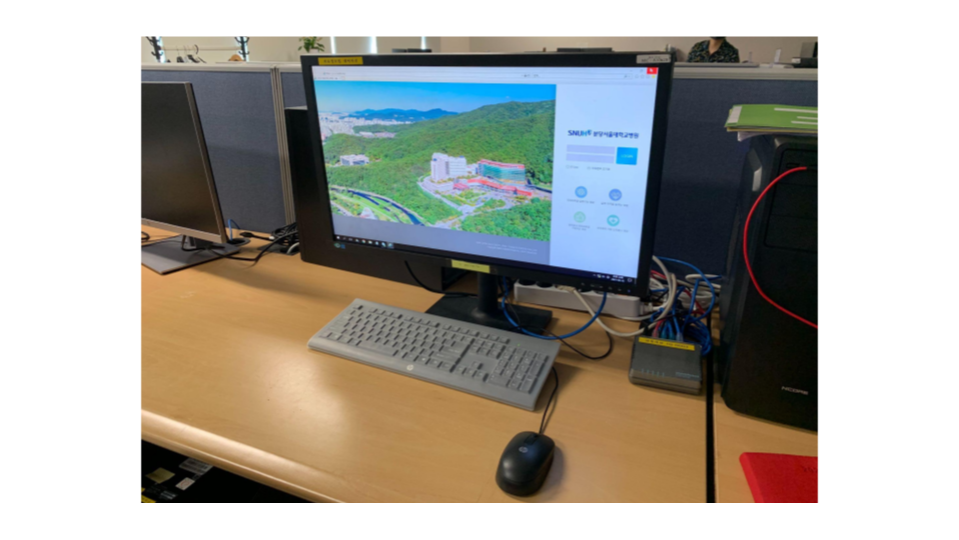
Monitoring unit with robotic process automation in Seoul National University Bundang Hospital. EMR: electronic medical record.

The RPA bot environment is set up to mirror that of a worker performing the target tasks. This setup includes a dedicated personal computer in an IT office, equipped with the RPA program, one virtual private network program, and 3 hospital system applications. To enable manual oversight, a monitor, keyboard, and mouse are also provided, allowing a manager to take control of the machine if necessary. A notable feature in security configuration is that RPA bots can use the same virtual private network program that workers use to access external networks. This capability distinguishes RPA from other automation technologies and thus necessitates additional security channels. Figure 1 shows the personal computer dedicated to RPA usage in the hospital office.

#### Robotic Process Automation Reports and Governance

**Figure 2:**
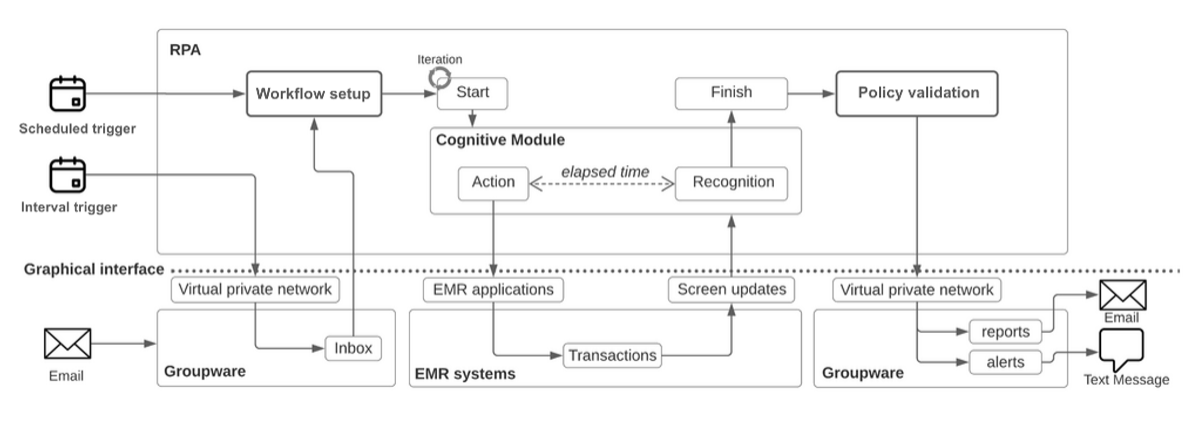
Process monitoring with robotic process automation.

RPA reporting processes and reactive governance were refined over several iterations. RPA bots could be activated either through a periodic scheduling system or upon receiving an email order from a supervisor. A single bot was responsible for executing dozens of business processes, estimating and reporting application performance while interacting with 18 modules within the systems. Each RPA business process was designed to mirror a critical business process within the hospital, affecting numerous other processes or significant clinical practices. The methodology for performance measurement was aligned with use perception. For instance, a request initiated by clicking a button on the interface was considered complete once a change was detected on a specific part of the screen. The performance metrics captured during this process were then recorded and documented in an Excel file. Based on the severity and abnormality of the data found, the RPA bot was also programmed to decide the recipients of email and SMS text message notifications, ensuring timely communication of potential issues. Figure 2 illustrates the process of system monitoring fulfilled by RPA agents.

**Figure 3:**
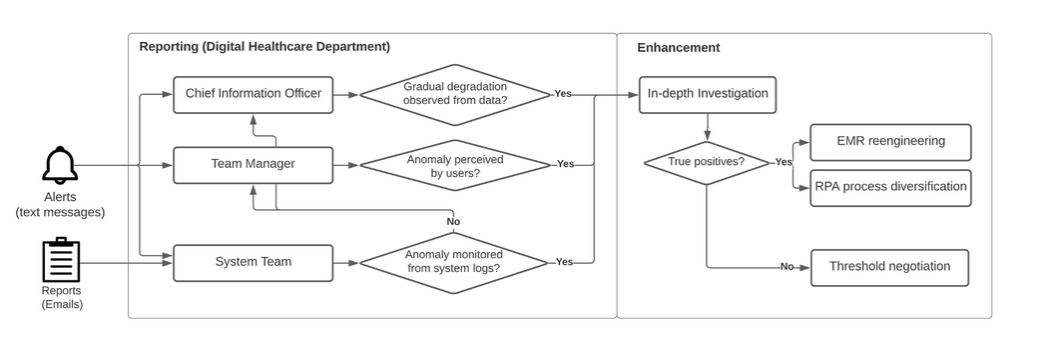
Organization’s reporting structure chart. EMR: electronic medical record; RPA: robotic process automation.

There was a strong consensus within the organization that the governance should capitalize on the RPA alerts by establishing a feedback loop to prevent the IT department from disregarding the findings due to excess false positives. This governance mechanism was designed to align with the hospital’s reporting structure. Mobile text messages were used to alert stakeholders about serious anomalies detected, while email served as the primary channel for routine reporting. To ensure oversight and prompt action, all alerts, if overlooked by middle managers and engineers, were escalated to the hospital’s Chief Information Officer. Figure 3 depicts the governance structure for reporting anomalies detected by RPA agents.

### Robotic Process Automation Monitoring Analysis

#### Overview

The goal of this study was to investigate the functionalities and advantages derived from implementing RPA in end-to-end monitoring within the health care sector. Given the innovative nature of this methodology in health care and the absence of quantitative benchmarks, a mixed methods approach was adopted. This approach aimed to assess the multifaceted value of RPA attributes, facilitating an understanding of how findings from quantitative and qualitative data converge [30].

#### Task-Oriented Robotic Process Automation Process Selection

The selection of RPA processes was deemed a critical aspect of the project to optimize the benefits of automation. Initially, 10 standardized processes at SNUBH, frequently used by clinicians, were identified. These encompassed 3 types of patient visits (inpatient, outpatient, emergency), as well as subsequent processes such as physician orders, common laboratory examinations, pharmacy visits, etc. These processes, while central to clinical practice, were noted for their significant latencies. RPA managers adopted a task-oriented approach in selecting the processes, aiming to ensure that the improvements or any degradations in performance would have a substantial effect on user experiences. As a result, functionalities across 56 pages in the main hospital application, which represent core interactions between the hospital’s main system application and its end users, were selected for automation.

#### Monitoring Data

The monitoring data comprised request-response duration data, alert records, and resolutions provided by the engineering team. For the selected functionalities, RPA agents performed the defined processes by navigating through 17 large service modules of the hospital information systems. During each interaction, primarily initiated by mouse clicks, the RPA agent awaited a detectable change on the screen and verified whether the expected execution had taken place. A screen change was deemed “adequate” if the image match rate exceeded 90% upon comparison with the standard screen where fetched data were expected to appear. After each execution, thresholds were consulted to decide if an alert should be issued.

RPA agents were scheduled 4 times daily from January 2021 to December 2024. A report was generated and sent to managers whenever the response time exceeded the set threshold. Over 3 years, more than 700 such events were reported to the management. Engineers worked alongside managers to pinpoint root causes and resolve the cases whenever feasible.

The data produced by RPA bots effectively highlighted the long-standing discrepancies between end users’ and engineers’ perceptions. Many recorded latencies showed unexpectedly large variances, underscoring the challenge of replicating the slowdowns experienced by end users. To quantify this issue, the gradual increase in latencies over several years was calculated, with processes showing significantly high variations subject to in-depth analysis. Indeed, most services were showing meaningful high fluctuations. In some complex cases, it was discovered that a module required a fundamental overhaul of its information structure, indicating that extensive refactoring was unavoidable.

#### Robotic Process Automation Manager Interview

The qualitative study targeted managers and system engineers within the IT department of SNUBH who had experience in developing or managing the RPA program. The objective was to uncover the core experiences to shed light on the role and effectiveness of RPA in the organization [31].

The sample size was directed by the research question [32], consisting of 8 managers and 7 engineers, each with at least 3 years of experience at the hospital. Purposeful samples were collected using snowball sampling until data redundancy or saturation was achieved, this point was identified when no new information emerged and was added to the findings extracted from previous interviews [33].

The analysis of participants’ experiences was primarily reductive as responses were broken down into smaller categories and later assembled to evaluate the measurement metrics. Initially, researchers thoroughly read the transcripts to immerse themselves in the texts. Subsequently, they coded the transcripts by identifying passages and meaning units that highlighted participants’ fundamental experiences of RPA. Of the 15 transcripts, a total of 392 significant meaning units were extracted. Finally, researchers revisited the transcripts alongside the measurement metrics to identify themes emerging from the meaning units.

## Results

### Overview

The results presented herein were derived from a dual approach that incorporates both quantitative and qualitative methods, combining the data generated by RPA robots with interviews conducted with RPA managers. The RPA bots, which have operated on predefined processes for over 3 years as introduced in the Methods section, have accumulated reports revealing significant insights. These insights, combined with the engineering team’s reactions to the reports, particularly highlight gaps in the user-perspective analysis that engineers have struggled to bridge. Multimedia Appendix 1 outlines the business processes monitored by RPA, including the domain, category, specific process steps, screen names, and threshold values (in seconds) for triggering alerts. Sample monitoring results are provided in Tables S1 and S2 in Multimedia Appendix 2.

The quantitative analyses in the sections mentioned below, “Uncovering User-Level Performance Fluctuations: RPA Bridges the Analysis Gap Between Users and Engineers,” “Holistic Incident Reporting: RPA Broadly Captures Diverse Causes of Issues,” and “RPA Manager Interviews: Best Practices are Calibrated Through Iterative Discussions,” focus on a comprehensive analysis akin to traditional end-to-end system monitoring. That is, the modern trend in software system monitoring emphasizes identifying core components and concentrating expertise in individual areas. However, the complexity of hospital information systems precludes a universally applicable strategy of compartmentalizing the structure. Unseen dimensions gradually undermine user experiences. The role of RPA agents has proven extremely effective in supplementing the existing monitoring governance, by performing important clinical processes “by hand.” The most unexpected result is the extensive coverage of RPA processes, which ensured system stability by identifying hidden errors after regular system deployments. This indicates a broader variety of reasons for the RPA agent to report incidents, offering a more accurate reflection of realistic user experiences.

These insights uncover long-standing challenges that users have implicitly faced for years. Sections mentioned below, “Quantifying ‘Real’ User Experience: RPA Detects Gradual Degradation in End-to-End Service Latencies” and “Uncovering User-Level Performance Fluctuations: RPA Bridges the Analysis Gap Between Users and Engineers,” detail how gradual performance degradation and inconsistencies have historically created disparities between user experiences and engineers’ performance metrics. Furthermore, the section “Holistic Incident Reporting: RPA Broadly Captures Diverse Causes of Issues” extends the discussion beyond mere performance tracking to include external influences and errors invoked by a deployment. While these issues should not happen theoretically, they become apparent only when combined with substantial real-world data, which sometimes contradicts the consistency assumed by engineers.

Finally, interviews with 20 managers, as discussed in the section “RPA Manager Interviews: Best Practices are Calibrated Through Iterative Discussions,” have further clarified the desired direction for RPA-driven monitoring systems. These interviews aimed to gather insights on both the perspective and progress of managers regarding RPA agents. The ability of RPA tools to provide a high-level system overview has quickly grown confidence in managerial support for RPA technology leading to more precise calibration of RPA tool usage. However, initial reservations about trust and data sensitivity persisted, highlighting areas where RPA agents are not effectively adaptable. This reveals practical limits that may vary depending on culturally diverse work ethics.

### Quantifying End-to-End Level User Experience: Robotic Process Automation Detects Gradual Degradation in End-to-End Service Latencies

Table 1 summarizes the latency of service calls, sampled from the interaction reports of RPA processes, which exhibited performance degradation accumulated over 3 years. The sampled service calls belong to 3 categories: infrastructure check, outpatient process, and inpatient process. This summary presents the first comprehensive evidence of end user experience, concretely demonstrating the long-term latency increase.

**Table 1:** Service latency analysis over years.

Robotic process automation process			Mean of latencies (seconds)						
Theme and service call			Year 2021		Year 2022		Year 2023		Increase
**Infrastructure**									
	Run the main client	4.9		5.1		6.5		1.6	
	Login to the main client	4.8		5.2		5.9		1.1	
	Run picture archiving and communication system	2		2		2.2		0.2	
	Login to picture archiving and communication system	9.2		9.5		12.2		3.0	
	Run groupware	3.5		3.6		3.8		0.3	
	Run virtual desktop infrastructure	12.9		13.4		13.2		0.3	
**Outpatient**									
	Outpatient listing	17.8		23.9		25		7.2	
	Outpatient selection	15		20.4		20.2		+5.2	
	Outpatient navigation	3.6		4.4		5		+1.4	
**Inpatient**									
	Inpatient listing	1.5		1.6		1.6		+0.1	
	Inpatient selection	2.3		2.8		3.2		+0.9	
	Inpatient navigation	2.5		2.3		2.5		+0	
	Nursing list	2.8		2.8		3		+0.2	
	Nursing list2	4		4.1		4.7		+0.7	

The table above lists three representative cases of processes and their selected functions executed by the RPA agent on various time frames on a daily basis. The changes in end-to-end latencies are recorded over 3 years. Among the most distinct cases, the outpatient process emerges as the most challenging to manage, displaying a clearly increasing trend in latency.

This straightforward result serves as critical evidence for the user experiences of many end users of the hospital systems who frequently complain about the system “feeling like” it is slowing them down. Engineering teams typically focus their monitoring efforts on the system’s principal components, such as database queries, server API calls, and network latencies. These measures are particularly effective in the early stages of the health care organization because improvements in these components typically lead to noticeable enhancements in user experiences. However, as the complexity of the hospital information system increases and services mature, hidden factors increasingly contribute to its performance degradation.

### Uncovering User-Level Performance Fluctuations: Robotic Process Automation Bridges the Analysis Gap Between Users and Engineers

Table 2 samples service calls that were highly ranked for showing significant variations. “Event Count” refers to the number of incidents reported by RPA agents over 3 years (2021-2023) due to exceeding their corresponding thresholds.

**Table 2:** Latency fluctuations.

Service call	Mean (SD)	Event count	Threshold
Nurse inpatient list	3.03 (0.55)	4	5
Nurse injection reservation	15.9 (1.6)	11	18
Health screening reservation	3.05 (0.52)	4	5
Examination results	6.94 (0.98)	19	8
Nutrition manage registration	2.66 (0.55)	7	5
Patient record viewer	4.24 (0.58)	112	5

One of the most significant contributions made by the RPA agent is the quantification of fluctuations in user experience. A significant number of the latencies recorded by the RPA agent demonstrated high SD, indicating that multiple incidents were detected and finally reported to hospital managers. For instance, the examination results function, which involves retrieving examination results for daily outpatients, exhibited notable instability, with an SD of almost 1 second against its usual latencies of 7 seconds. This function encompasses multiple sub-fetching calls, updated weekly, making it difficult for individual performance testing to ensure stability from a holistic perspective, despite latencies generally falling within acceptable thresholds according to engineers’ environments.

This evidence of a broad spectrum of latencies is particularly valuable in debates between users and engineers struggling with the lack of reproducibility of the delayed system response users experience. Even small bugs are difficult to pinpoint from a user’s perspective, which is not their primary role in the organization. Expanding the scope to performance, many diagnoses suggested by users were considered insufficient as engineering reports were consistently indicating component-level monitoring within 0.01-second intervals. However, such evidence collected by engineers has failed to capture the true user experience.

Indeed, the complex nature of these systems, with their interdependencies and the frequent updates of components, can obscure the root causes of performance fluctuations. As systems evolve and expand over decades, new features introduced outside the existing framework often inadvertently introduce inefficiencies. Moreover, even if all individual factors are efficiently manageable, the dynamic nature of hospital operations, including variable patient loads and the unpredictable demand for different functions, exacerbates these challenges.

### Holistic Incident Reporting: Robotic Process Automation Broadly Captures Diverse Causes of Issues

Table 3 groups events reported by RPA agents based on the cause of the occurrence and the response to each event. Initially, the focus was solely on detecting query degradation events. However, as monitoring progressed, the scope of categories expanded. Identifying erroneous deployment events proved particularly effective in preventing significant operational disruptions in the hospital. Query degradation is focused on specific query inefficiencies, while performance inconsistency encompasses broader, variable performance fluctuations across the system. Erroneous deployment: errors detected immediately after deployment due to misconfigurations or incomplete processes. Bad deployment: performance degradation observed postdeployment caused by insufficient planning or testing.

**Table 3:** Incident cases.

Event type	Cases	Reasons	Resolutions
Erroneous deployment	9	An error was detected immediately following a software deployment.	Resolve the identified error.
External program issues	5	External programs caused issues (network, authority, error, etc)	Resolve the identified issue.
Bad deployment	3	Performance deteriorated noticeably right after software deployment.	Optimize performance post-deployment.
Query degradation	3	Inefficiencies in query execution became evident with increasing data volume.	Enhance query efficiency and performance.
Performance inconsistency	660	Query response times fluctuate with daily data volume changes.	Develop a strategy for structural improvements.

One of the unexpected yet invaluable contributions of the RPA agent has been its holistic approach to testing. Originally aimed at measuring system performance from the users’ perspective and quantifying their experiences, the scope of the RPA agent’s coverage extended far beyond these initial objectives. For example, in the case of erroneous deployment, the RPA agent identified errors immediately following regular software deployments, with all 9 reported cases attributable to the intricate complexities of the hospital information systems. Often, modifications in one module inadvertently trigger errors in another, through the information flow of real data. These are the types of issues typically addressed by application-level testing, yet designing a comprehensive experiment often eludes the grasp of individual engineers.

Furthermore, the RPA’s goal cannot solely be the exhaustive identification of errors, which is extremely difficult to achieve. Instead, the true value of the automated agent’s reports lies in ensuring that common processes (daily tasks performed by thousands of employees for tens of thousands of patients) remain unaffected. This precaution helps prevent incidents from escalating into broader performance issues that could impact the entirety of hospital operations.

Second, the second row in the table illustrates that, while the RPA processes operate in the same environment as users, they are susceptible to the influence of external, peripheral, and temporary system components. Notably, one incident was reported due to a memory leak from an embedded external program. The integration of third-party applications or web-based services is sometimes overlooked due to their perceived minimal impact on the overall system. However, the trend toward incorporating a diverse array of software products has transformed the system into a complex assembly of heterogeneous components that interact with one another, potentially affecting major processes through their services.

Finally, as detailed in this section, the identification of 660 cases of performance inconsistencies has prompted the engineering team to strategize toward stabilizing the end-to-end latencies of major practices, including the outpatient management process. This initiative underscores the RPA agent’s crucial role in addressing diverse issues; it not only detects immediate issues following regular deployments but also facilitates long-term improvements to enhance system reliability and user satisfaction.

### Robotic Process Automation Manager Interviews: Best Practices Are Calibrated Through Iterative Discussions

The qualitative data collected through interviews illuminated how the adoption of RPA impacted the system and the organization. A central point of discussion was “Which processes should be monitored?” leading to a more explicit understanding of the pros and cons of adopting RPA bots.

Key phrases extracted from the interviews were then categorized into 5 clusters aligned with core themes: understanding of RPA, prediction capability, trust in robots, downsides of cognitive agents, governance facilitator, and the RPA epiphany. These encodings provide concrete descriptions of the abstract themes, underpinned by sampled phrases.

Table 4 categorizes the primary advantages of implementing RPA as identified by managers, with specific themes and corresponding coding. Sample phrases from interviews illustrate the practical impact of these benefits on process efficiency, cross-departmental cooperation, and governance within the organization.

**Table 4:** Summary of robotic process automation benefits based on managerial feedback.

Themes (pros) and coding		Sample phrases
**Ease of understanding**		
	Simplicity of processes Repetition of processes Well-defined processes	“The simplicity is that the task does not require human judgment and is defined by a clear business rule”
**Cooperation invoking**		
	Cross-departmental collaboration Joint software update tests	“...Moreover, we can impose an RPA^a^ bot on all tasks that were covered by 16 managers from different departments”
	Responsive governance	“...However, the bot now delivers an SMS or email to each manager responsible for each process. Speed has always been an issue, and now it is working....”
**Governance facilitation**		
	Evolving thresholds Engineer-user negotiation	“...I argued that the right way is to focus more on whether it hampers the process for the user. Thresholds were set in such a conservative manner. ”
	Enhancing business interoperability	“...And then another email can re-trigger RPA bots to confirm the resolution of the issue.”

^a^RPA: robotic process automation.

A majority of managers highlighted the benefits of adopting this new technology as a reliable system monitoring and comprehensive testing service. First, the ease of understanding RPA was seen as a significant advantage, offering managers user-friendly methods to interpret processes focused primarily on clinical practices. Managers appreciated the intuitive and understandable process definitions, clearly outlined on the system’s GUI.

Second, RPA to foster departmental cooperation was seen as ideal for addressing the challenges posed by hospital information systems, which are often divided into disjointed components. From the UI perspective, with which the RPA agent interacts to simulate human behaviors without assuming any engineering team affiliation, one RPA process can interact with up to a dozen components, identifying cross-departmental issues arising from information inconsistencies. Addressing one issue typically requires collaboration among engineers from multiple departments to determine its root cause.

Third, the RPA agent acts as a governance facilitator, enabling more realistic and evidence-based negotiations between engineers and users. The previously dominant engineer-driven metrics proved inadequate for sustaining effective discussions on system performance management. However, as indicated in the sections above, the focus could be shifted toward quantifying user experiences, thereby identifying how efforts should be allocated for process improvements.

Table 5 summarizes managerial feedback on the drawbacks of RPA, including trust issues, cognitive limitations, and insightful epiphanies from its application.

**Table 5:** Managerial insights on robotic process automation challenges.

Themes (cons) and coding			Sample phrases
**Trust issues with the robots**			
	Recognition accuracy Handling of sensitive clinical tasks	“...The system suddenly alerts me of OCR failures which was the case until yesterday. We cannot allow its applications to patient-related tasks if it isn’*t* 100% consistently guaranteed.”	
**Downsides of cognitive agents**			
	Ambiguous state definition	“...The current configuration checks whether the hospital logo is floating to decide if the website has loaded. However, the image server often fails while the other functions are completely working.”	
	Unexpected maintenance costs	“...Yes, robotic process automation recognizes a shape as people do, but that depends on the context. The bot can find a red button after being relocated but it won’t find the button when color or text changes.”	
**Deriving robotic process automation epiphanies**			
	Persuasive demonstration Generation of personalized ideas	“...and I felt ‘Oh, that’s what this is doing’. After that, the application seemed to be limitless...”	

However, the accumulated experiences have not repositioned RPA bots as a universal solution applicable to all clinical processes. First, the most sensitive issue relates to the scope of information that RPA agents handle. Many major processes involving complex and critical clinical information require RPA agents to manipulate information states. Although all state-modifying operations are designed to work with dummy data generated exclusively for the RPA agent, unpredictable incidents can arise from misinterpreting commands, affecting data on the production server. Ironically, as AI-based decisions become more complex and human-like, the likelihood of errors increases, mirroring those made by inexperienced workers. This risk has deterred managers from extending the monitoring scopes to clinically sensitive services, which are deemed crucial areas for robotic coverage.

Second, contrary to expectations, RPA agents, as cognitive agents, lack flexibility in varying situations. OCR-based recognitions depend on hints from managers, which may become outdated due to system maintenance. Since RPA processes involve complex commands based on OCR recognition logic (eg, detecting some pop-ups) there is a greater chance of encountering unadaptable modifications. For example, an unrelated pop-up appearing during a process, easily dismissed by a human tester, poses a challenge for an RPA agent interpreting it as an incorrect message response. These scenarios have led to unexpected maintenance costs and increased frustration due to inconsistencies, making the unambiguous definition of UI interactions a significant challenge for managing the vast number of RPA agents.

Third, the “RPA epiphany” has not been universally shared. Developing a productive RPA process requires the cooperation of clinicians and other hospital staff, yet reactions to the same approach can vary. Some employees fear job loss to highly efficient robots, while others see it as creating a burdensome new task, compelling them to manage the tedious robot postinitialization. Both perspectives hold some validity, necessitating extensive discussions to expand use cases.

## Discussion

### Study Summary

The effectiveness of RPA in monitoring EMR systems was measured through latency data and interviews. In response to the demand of end users who have sought a new indicator reflecting the quantification of their experiences, RPA processes were carefully chosen to monitor the essential clinical processes containing information-heavy user tasks. The latency data collected through the monitoring process proved that indeed the comprehensive perspective of RPA bots serves as the bridge valuing the cross-disciplinary efforts of users and engineers. Finally, the qualitative interview revealed notable characteristics of RPA that affected the EMR management organization to reform the governance, and highlighted RPA bots, as a new cognitive agent resembling humans, having pros and cons.

Which process should be selected for the monitoring tasks? This simple question has been an important topic ever since the first emergence of BPM [34] because most automation project lifecycles started from process identifications. With a prevalent top-down approach, the start of a project was focused on business goals [35] and then spread to subordinate departments and users. However, such an approach was often attributed to the failure of recent RPA implementations, motivating studies to standardize and quantify process selections in RPA projects [36,37]. The strategy used by managers in the SNUBH project involved iteratively identifying the most common components impacting real-world practices, with a focus on using RPA agents to simulate the tedious testing tasks performed by end users. This approach, though simple, proved to be exceptionally effective. The collected reports provided insights into end user experiences that were among the closest to any similar efforts done to gather data for the same purpose.

Previous studies in APM have been challenged by significant fluctuations due to user-level performance measurements, requiring extensive expert analysis [11,12]. However, the integration of RPA bots, aimed at mirroring the “real” user experience as closely as possible, transforms the apparent downside of APM into an advantage. The delays observed in Table 1, although mostly under 5 seconds, may not significantly impact end user experience on an individual basis. However, the cumulative effect of these small delays over extended working hours could influence overall productivity. Furthermore, understanding the root cause of such delays is crucial. Delays resulting from natural database growth might be considered acceptable, while those stemming from other factors should prompt further investigation. RPA plays a pivotal role in identifying and quantifying these delays, enabling management to assess whether they fall within acceptable performance thresholds and take corrective actions when necessary. The RPA project team prioritized ensuring the usability of the most critical hospital processes to enhance end user experiences. This approach is entirely user-centric, lacking any scientific purpose or methodology, stemming from the recognition that the system’s complexity prevents engineers from maintaining a comprehensive view of the services. User-driven process monitoring accurately reflects true priorities, preventing engineers from becoming overly fixated on component-level details.

The observed increase in task completion times, as shown in Table 1, aligns with the natural growth of the database over time, which can contribute to delays in end-to-end service. In addition, the system management (SM) team at the study hospital develops and implements approximately 700 new programs annually to enhance EMR operational efficiency. These frequent updates can also impact service performance. The integration of RPA proved beneficial in detecting such delays, enabling targeted analyses of their root causes. This facilitated database tuning by database administrators and prompted the SM team to review and optimize newly implemented program code, ultimately improving system performance and ensuring operational continuity.

Moreover, the monitoring process can still be initiated by engineers, serving as a starting point for identifying key areas for improvement. Performance degradation data collected by RPA agents involve multiple factors, necessitating cross-departmental collaboration, which can lead to inefficient practices in identifying root causes. Indeed, most cases required several hours to days to identify potential engineering factors. However, there is a consensus that these factors are of the highest priority for affecting the real-time practices of end users, and the performance standards to be maintained can be relatively easily negotiated. On the other hand, the consistent increase in system complexity has made component-centric analysis and improvements less impactful, as they no longer directly enhance user experiences. RPA agents act as proxy users, emphasizing the most critical information flows that the system must maintain smoothly. The low standard deviation observed in Table 2 indicates minimal fluctuation in delay times, suggesting stability. However, this stability also reveals that delays are consistently recurring rather than sporadic. Such persistent delays, even if small, can accumulate over time and impact overall system efficiency. This underscores the importance of addressing not just large fluctuations but also ongoing, minor delays. RPA’s capability to monitor and report these patterns enables targeted optimization efforts, helping to mitigate their long-term effects on system performance and user experience.

Interview themes unveiled the complex ways in which the organization leverages new technology. RPA, acting as a cognitive agent, introduced a novel approach to automation integration. RPA bots remained “lightweight,” effortlessly accessing functions by overlaying the interface of target EHR applications [38]. These features made technical barriers minimal, allowing RPA managers to implement RPA logic with their extensive knowledge of application-level practices without frequently consulting EHR engineers during the project. The speed of integration was accelerated by the straightforward development process and the flexible recognition capabilities supported by RPA.

However, as mentioned in the Method section, unforeseen drawbacks of RPA emerged due to its reliance on UI-based interactions. These included a vast number of screen change recognitions, and although RPA was adaptable enough to approximate detections, such as locating a button with a specific phrase, modifications to the UI itself exceeded the scope of its flexibility. Consequently, UI elements, being primarily graphical and perceived as less critical to service logic, now require a shift in perception and further study for extended and reliable RPA use.

An incident where an engineer identified the hospital logo as a weak point of the configuration, which defined the loaded groupware web page, illustrates this challenge. The logo's reliance on a content delivery network service, which frequently failed, underscored a common misunderstanding of UI behaviors. In previous studies, this issue has been explored in ontology-oriented UIs, which advocate for the declaration of high-level models and their translation into program UIs [39]. An ontology, in this context, is defined as “a logical theory comprising a formal vocabulary” [40] that supports UIs, ensuring precise and consistent conceptual organization of the UI [41]. Therefore, developing an intermediate ontological model of the UI to support RPA projects could be a crucial preliminary step.

Observation of the RPA adoption led to an evaluation of the organizational-level contribution of RPA to the complexity management of EHR systems. Meaningful subjects included threshold evolution, responsive governance, and business interoperability facilitation. Threshold evolution was the unanticipated practice where engineers and supervisors negotiated thresholds of alerting performances to cut off false positives while preserving enough predictive qualities.

Beyond performance monitoring, SNUBH supervisors emphasized that the RPA project significantly enhanced responsive governance. RPA acted as a conduit between users and machines, transforming subjective empirical experiences into objective, meaningful data. From this perspective, RPA emerged as an ideal evidence collector, silently tracking fluctuations in system performance without the burden of its repetitive, tedious tasks.

In terms of business interoperability, organizational responsiveness was notably improved through the use of RPA bots, which served as digital messengers at the application level. Users could send requests to and receive reports from RPA bots via email, mirroring the function of chatbots—computer programs designed to simulate human conversation. Gradually, RPA bots became part of the workforce team, communicating through standard messaging channels and enhancing end user satisfaction [42].

### Core Implications

This study’s outcomes will enlighten decision-makers about the capabilities of RPA in evaluating business process performance, with a specific focus on simulating user activities on complex EHR systems. Initially, the adoption of RPA for monitoring EHR systems was anticipated to surge rapidly due to its direct and intuitive development procedures. However, the revealed end user experience was remarkably poor: the study uncovered significant variations in the latencies of crucial common service calls. Such monitoring data can be regarded as the definitive “ground truth” of end user experiences, fostering constructive discussions between users and engineers.

Nonetheless, challenges like UI-driven maintenance issues and the complexity of interpreting results complicate ongoing quality control efforts. To mitigate these issues, there should be the establishment of standardized, iterative practices aimed at judiciously identifying areas deserving daily attention from RPA agents. Furthermore, psychological resistance could decelerate RPA adoption in health care settings. This reluctance stems from the cognitive capabilities of RPA bots, which closely mimic tasks traditionally performed by human employees, raising concerns about their potential impact on the job market in the industry.

### Future Directions

RPA naturally overcame the shortcomings by providing event-driven configurations based on cognitive analysis of the screen changes. However, some questions recognized in this study should be answered to aid in the success of future RPA projects.

Could RPA bots entirely replace traditional GUI-based testing frameworks? After stabilizing RPA bots to detect process performance degradations, the EMR management team found that RPA bots are eventually fulfilling an advanced GUI-based testing role by resolving the following challenges. Most GUI tests showed target application-dependent performance measurements [43] and object recognition techniques [44] that limited their usage across a large system. Also, GUI testing automation suffered a “temporal synchronization problem” arising from timer-based process execution, limiting flexibility for any latencies [45,46]. Recent studies seem to recognize the potential benefits of RPA in the field [47,48].

How can system complexity be managed after RPA adoptions? This question is directly connected to the practice in the project but, as a goal of this study, also applies to future RPA implementation projects. Since RPA sits upon the other systems, it creates another layer of complexity in the automation. It is anticipated that most RPA projects will devise a monitoring protocol as part of the automation to maintain performance. Furthermore, RPA is considered slow by certain companies because its front-end level execution is inferior to that of back-end systems [49], and performance measurement is difficult, so overall system complexity after integration has to be considered in the decision to adopt RPA.

How can RPA build trust among users of EHR systems? A common misunderstanding about RPA is that the automation is accurate simply because the implementation is easy and intuitive for users. However, as revealed in this study, users were skeptical in at least 2 cases. First, the mismatch between GUI and user intent remained unresolved. Ontological models were suggested to match the domain behind GUI. The growing importance of such models is due to their practical usage, which allows workers and automation engineers to seamlessly accept the domain that supports the GUI. Second, contrary to this pragmatic view, user trust in cognitive recognition was not sufficient even though failure rates were low. User trust issues in automation are not new and require time because EHR users tend to be more conservative about this.

How can RPA harmonize with organizations and their governance? It has been acknowledged that a common mistake in treating RPA is to limit its role as a simple piece of software rather than as a foundation for leading digital transformations [50]. Therefore, RPA governance should be an integral aspect of the implementation of RPA so that scalable cross-departmental use is realized. The components of RPA governance may include process identification and prioritization, distribution of RPA bot responsibility, RPA knowledge management, and reward systems [51].

### Study Limitations

The limitations of this study lie in the practical constraints of the SNUBH project. First, this study only applied RPA to a single testbed. Significant considerations were required to make the business process performance monitoring model generalizable. However, the project results and other references imply that expanding and transferring RPA implementation is relatively straightforward once demonstrated to work for one scenario, unlike frameworks relying on specific technology stacks. This low degree of system dependency allows RPA modules to be adapted to similar systems.

A second limitation is that RPA was not configured to cover the diverse infrastructural environments and actual end user experiences. End users working in SNUBH have divergent computer environments and application usage patterns. This has raised criticism as to whether RPA bots properly contribute to complexity management when they operate in such a controlled environment. A compromise had to be made by setting the computer running RPA bots to mirror the computers used by general end users as closely as possible. This decision was also influenced by the fact that the initial goal of this project was not to measure the user experience comprehensively but to identify performance outliers where there was a noticeable change in business processes.

Third, the level of understanding of RPA among the interview participants was inconsistent, which was eventually accepted as part of their characteristics. The study intended to measure the differences from existing methodologies, but the perception of RPA was more varied than expected. For example, some engineers focused on RPA’s effects as an automation tool rather than understanding it as a source of modifying the governance structure of the organization. Furthermore, some supervisors lacked a clear understanding of the technical definition of RPA.

Fourth, implementing RPA in diverse health care settings presents additional challenges not addressed in this study. Variations in organizational structures, technological systems, and regulatory environments can significantly influence the success of RPA adoption. For instance, RPA integration may face difficulties in environments with legacy systems or fragmented workflows. In addition, scaling RPA across larger institutions often requires substantial resource allocation and staff training, which may limit its applicability. Compliance with data protection and privacy regulations adds another layer of complexity, demanding meticulous planning to ensure alignment with legal standards. Future research should investigate these challenges to provide a more comprehensive understanding of RPA’s role in diverse health care contexts.

## Appendix

Multimedia Appendix 1 Target business processes and example data.

Multimedia Appendix 2 Target business processes and example data and sample results of system monitoring.

## Abbreviations

AI: artificial intelligence
APM: application performance monitoring
BPM: business process management
EHR: electronic health record
EMR: electronic medical record
GUI: graphical user interface
HIPAA: Health Insurance Portability and Accountability Act
IoT: Internet of Things
IRB: institutional review board
RPA: robotic process automation
SM: system management
SNUBH: Seoul National University Bundang Hospital
UI: user interface

## References

[1] Davenport T, Kalakota R (2019). The potential for artificial intelligence in healthcare. Future Healthc J.

[2] Wang F, Preininger A (2019). AI in health: state of the art, challenges, and future directions. Yearb Med Inform.

[3] Zeadally S, Siddiqui F, Baig ZA, Ibrahim AM (2019). Smart healthcare. PRR.

[4] Alamri A (2018). Ontology middleware for integration of IoT healthcare information systems in EHR systems. Computers.

[5] Zhang R, Liu L (2010). Security models and requirements for healthcare application clouds.

[6] Plsek PE, Greenhalgh T (2001). Complexity science: the challenge of complexity in health care. BMJ.

[7] Tutty MA, Carlasare LE, Lloyd S, Sinsky CA (2019). The complex case of EHRs: examining the factors impacting the EHR user experience. J Am Med Inform Assoc.

[8] Greenhalgh T, Papoutsi C (2018). Studying complexity in health services research: desperately seeking an overdue paradigm shift. BMC Med.

[9] Kannampallil TG, Schauer GF, Cohen T, Patel VL (2011). Considering complexity in healthcare systems. J Biomed Inform.

[10] Abbott PA, Foster J, Marin HDF, Dykes PC (2014). Complexity and the science of implementation in health IT--knowledge gaps and future visions. Int J Med Inform.

[11] Molyneaux I (2014). The Art of Application Performance Testing: From Strategy to Tools.

[12] Heger C, van Hoorn A, Mann M (2017). Application performance management: state of the art and challenges for the future.

[13] Bate P, Robert G (2007). Bringing User Experience to Healthcare Improvement: The Concepts, Methods and Practices of Experience-based Design.

[14] Taulli T (2020). The robotic process automation handbook. A Guide to Implementing RPA Systems.

[15] Primer A (2015). Institute for Robotic Process Automation.

[16] Aguirre S, Rodriguez A (2017). Automation of a business process using robotic process automation (RPA): a case study.

[17] Huang F, Vasarhelyi MA (2019). Applying robotic process automation (RPA) in auditing: a framework. International Journal of Accounting Information Systems.

[18] Kaya CT, Turkyilmaz M, Birol B (2019). Impact of RPA technologies on accounting systems. Muhasebe ve Finansman Dergisi.

[19] Moffitt KC, Rozario A, Vasarhelyi MA (2018). Robotic process automation for auditing. Journal of Emerging Technologies in Accounting.

[20] Belden JL, Grayson R, Barnes J (2009). Defining and testing EMR usability: principles and proposed methods of EMR usability evaluation and rating. Information Experience Laboratory Publications (MU).

[21] van der Aalst WMP, Bichler M, Heinzl A (2018). Robotic process automation. Bus Inf Syst Eng.

[22] Muehlen MZ, Ho DTY (2006). Risk management in the BPM lifecycle.

[23] Lenz R, Reichert M (2007). IT support for healthcare processes – premises, challenges, perspectives. Data & Knowledge Engineering.

[24] Musa MA, Othman MS (2016). Business process reengineering in healthcare: literature review on the methodologies and approaches. RES.

[25] Buttigieg S, Dey PK, Gauci D (2016). Business process management in health care: current challenges and future prospects. IEH.

[26] Flechsig C, Lohmer J, Lasch R (2019). Realizing the full potential of robotic process automation through a combination with BPM. Logistics Management.

[27] Dumas M, La RM, Mendling J, Reijers HA (2013). Fundamentals of Business Process Management.

[28] Kowall J, Cappelli W (2012). Magic quadrant for application performance monitoring. Gartner Research ID G.

[29] Ahmed T, Bezemer C, Chen T, Hassan A, Shang W (2016). Studying the effectiveness of application performance management (APM) tools for detecting performance regressions for web applications: an experience report.

[30] Venkatesh V, Brown SA, Bala H (2013). Bridging the qualitative-quantitative divide: guidelines for conducting mixed methods research in information systems. MISQ.

[31] Neubauer BE, Witkop CT, Varpio L (2019). How phenomenology can help us learn from the experiences of others. Perspect Med Educ.

[32] Pope C, Ziebland S, Mays N (2000). Qualitative research in health care. Analysing qualitative data. BMJ.

[33] Marshall MN (1996). Sampling for qualitative research. Fam Pract.

[34] Lee RG, Dale BG (1998). Business process management: a review and evaluation. Business Process Management Journal.

[35] Cho C, Lee S (2011). A study on process evaluation and selection model for business process management. Expert Systems with Applications.

[36] Wanner J, Hofmann A, Fischer M, Imgrund F (2019). Process selection in RPA projects-towards a quantifiable method of decision making.

[37] Soybir S, Schmidt C, Liermann V, Stegmann C (2021). Project management and RPA. The Digital Journey of Banking and Insurance, Volume I: Disruption and DNA.

[38] Willcocks LP, Lacity M, Craig A (2015). The IT function and robotic process automation.

[39] Kleshchev A, Gribova V (2003). From an ontology-oriented approach conception to user interface development.

[40] Guarino N (1998). Formal ontologies and information systems.

[41] Shahzad S, Granitzer M, Helic D (2011). Ontological model driven GUI development: user interface ontology approach.

[42] Anagnoste S (2018). Robotic automation process - the operating system for the digital enterprise. Proc Int Conf Bus Excell.

[43] Adamoli A, Zaparanuks D, Jovic M, Hauswirth M (2011). Automated GUI performance testing. Software Qual J.

[44] Alégroth E, Feldt R, Ryrholm L (2014). Visual GUI testing in practice: challenges, problemsand limitations. Empir Software Eng.

[45] Borjesson E, Feldt R (2012). Automated system testing using visual GUI testing tools: a comparative study in industry.

[46] Yuan X, Cohen MB, Memon AM (2009). Towards dynamic adaptive automated test generation for graphical user interfaces.

[47] Cernat M, Staicu A, Stefanescu A (2020). Improving UI test automation using robotic process automation.

[48] Heiskanen A (2021). Robotic process automation in automated GUI testing of web applications.

[49] Osman CC (2019). Robotic process automation: lessons learned from case studies. IE.

[50] Willcocks L, Hindle J, Lacity M Executive Res Rep, Knowl Capital Partners, USA, Tech Rep. Keys to RPA success.

[51] Smeets M, Erhard R, Kaußler T (2021). Introduction of RPA governance. Robotic Process Automation (RPA) in the Financial Sector: Technology - Implementation - Success For Decision Makers and Users.

