# Supplementary material for: Applying Robotic Process Automation to Monitor Business Processes in Hospital Information Systems: Mixed Method Approach
Source: JMIR Med Inform. 2025 Mar 7;13:e59801. doi: 10.2196/59801 (PMC11928770; doi:10.2196/59801)
Supplement: Multimedia Appendix 1 [file medinform_v13i1e59801_app1.docx]

**Target business processes and example data**

List of monitoring items with RPA. This table lists the business processes monitored by RPA, detailing the domain, category, specific process steps, screen names, and the threshold values in seconds for triggering alerts.

| Domain | Category | Sub-category 1 | Sub-category 2 | Screen No | Screen name | Threshold value for alert (second) |
| --- | --- | --- | --- | --- | --- | --- |
| BESTCare home | | | | **1** | BESTCare home | **8** |
| BESTCare Login | | | | **2** | BESTCare Login | **8** |
| Physician & Nurse | Physician Main | Physician Order | Patient Management | **3** | Patient Management – Outpatient | **25** |
|  |  | Physician Order | Patient Management | **4** | Patient Management – Outpatient | **25** |
|  |  | Physician Order | Patient Management | **5** | Patient Management – Outpatient | **7** |
|  |  | Physician Order | Patient Management | **6** | Patient Management – Inpatient | **5** |
|  |  | Physician Order | Patient Management | **7** | Patient Management – Inpatient | **5** |
|  |  | Physician Order | Patient Management | **8** | Patient Management – Inpatient | **6** |
|  |  | Physician Order | Patient Management | **9** | Patient Management – ER patient | **5** |
|  |  | Physician Order | Patient Management | **10** | Patient Management – patient search | **10** |
|  |  | Medical Record | Record Retrieval | **11** | Physician Record Retrieval | **20** |
|  |  | Physician Order | Physician Order Management | **12** | Order Retrieval | **5** |
|  |  | Physician Order | Physician Order Management | **13** | Order Issuing | **7** |
|  |  | Physician Order | Patient Registration | **14** | Diagnosis Registration | **5** |
|  |  | Medical Record | Medical Record Viewer | **15** | Medical Record Viewer | **5** |
|  | Nursing Main | Nursing Main | Patient List | **16** | Nursing Main – Patient List – Inpatient | **5** |
|  |  | Nursing Main | Patient List – Patient Retrieval | **17** | Nursing Main – Patient List – Inpatient | **6** |
| Indirect Care | Radiography | Registration | Check in | **18** | Imaging Test - Registration and Execution - Test Registration | **5** |
|  |  | Registration and Execution | CT Test Execution Registration | **19** | CT Test Execution Registration | **5** |
|  | Department of Diagnostic Laboratory Medicine | Registration Management | Preparation Management | **20** | Patient Blood Collection | **5** |
|  | Pharmacy | Pharmacy Information | Drug Master | **21** | Basic Drug Information | **5** |
|  |  | Outpatient Dispensing | Medication Management | **22** | Outpatient Prescription Audit | **5** |
|  |  | Inpatient Dispensing | Prescription Management | **23** | Inpatient Dispensing Order/Audit | **5** |
|  | Medical Records | Medical Record Copy Issuance Management | Copy Issuance | **24** | Medical Record Copy Printing | **5** |
|  | Imaging Test | Test Inquiry | Test Inquiry | **25** | Test Result Inquiry | **30** |
|  | Health Promotion Center | Health Certificate Reservation Management | Health Certificate Reservation Management | **26** | Health Certificate Reservation Management | **5** |
|  |  | Health Certificate Execution Management | Test Registration and Performance | **27** | Test Registration and Performance | **5** |
|  |  | Judgment Result Management | Judgment Result Management | **28** | Judgment Result Management | **5** |
|  | Food Nutrition | Nutrition Management | Clinical Nutrition Management | **29** | Nutrition Management Referral Reception | **5** |
|  |  | Nutrition Management | Clinical Nutrition Management | **30** | Inpatient Nutrition Search | **6** |
|  | Radiation Oncology Treatment | Information Management | Patient Information Management | **31** | Nurse Room/Patient Inquiry by Reception Treatment Room | **18** |
|  |  | Information Management | Patient Information Management | **32** | RTP Patient Inquiry | **5** |
|  | Rehabilitation Therapy | Information Management | Patient Management | **33** | Therapy Appointment Inquiry | **7** |
|  |  | Information Management | Statistics Management | **34** | Rehabilitation Statistics | **29** |
|  |  | Information Management | Treatment Management | **35** | Test Result Registration | **8** |
|  | Special Test Department | Test Management | Reservation and Registration Management | **36** | Test Registration | **5** |
|  |  | Test Management | Test Execution Management | **37** | Test Execution | **10** |
|  |  | Test Management | Reservation and Registration Management | **38** | Reservation Inquiry | **6** |
|  |  | Test Management | Interpretation Result Management | **39** | Interpretation Result Registration | **6** |
|  | Pathology | Registration Management | Registration Management | **40** | Other Department Sample Registration | **10** |
|  |  | Registration Management | Registration Management | **41** | Operating Room Sample Registration | **14** |
|  |  | Results Management | Results Management | **42** | Registration Status Inquiry | **5** |
|  |  | Results Management | Results Inquiry | **43** | Pathology Diagnosis Result Inquiry | **5** |
| Medical Affairs | Medical Affairs | Patient and Appointment Management | Outpatient | **44** | Outpatient Appointment Registration | **8** |
|  |  | Patient and Appointment Management | Inpatient Appointment | **45** | Inpatient Room Assignment Registration | **5** |
|  |  | Patient and Appointment Management | Emergency Patient | **46** | Emergency Room Discharge Patient and Status | **5** |
|  |  | Medical Fees Management | Medical Fee Management(Outpatient/Emergency) | **47** | Outpatient Medical Fee Payment | **10** |
| Insurance | Insurance | Inpatient Review Management | Preliminary/Inpatient Review | **48** | Preliminary/Inpatient Review | **5** |
|  | Insurance | Review Reduction Management | Review History Management | **49** | Review Result Inquiry | **5** |
| PACS | Login | Initial Access | PACS Initial Access | **50** | PACS Initial Access | **5** |
|  | Login | Login | PACS Login | **51** | PACS Login | **19** |
|  | Worklist Inquiry | Worklist Inquiry | PACS Worklist Inquiry | **52** | PACS Worklist Inquiry | **5** |
| Groupware | Login | Initial Access | Groupware Initial Access | **53** | Groupware Initial Access | **5** |
|  | Login | Login | Groupware Login | **54** | Groupware Login | **7** |
| VDI | Login | Login | VDI Initial Access | **55** | VDI Initial Access | **5** |
|  | Login | VM Access | VDI Login | **56** | VDI Login | **16** |
